# Characterization of SARS-CoV-2 antibodies in human milk from 21 women with confirmed COVID-19 infection

**DOI:** 10.1038/s41390-022-02360-w

**Published:** 2022-11-25

**Authors:** Lars Bode, Kerri Bertrand, Julia A. Najera, Annalee Furst, Gordon Honerkamp-Smith, Adam D. Shandling, Christina D. Chambers, David Camerini, Joseph J. Campo

**Affiliations:** 1 Departments of Pediatrics, University of California, San Diego, La Jolla, CA 92093 USA; 2 Larsson-Rosenquist Foundation Mother-Milk-Infant Center of Research Excellence (MOMI CORE), University of California, San Diego, La Jolla, CA 92093 USA; 3 Antigen Discovery Inc. (ADI), Irvine, CA 92618 USA; 4 Herbert Wertheim School of Public Health and Longevity Science, University of California, San Diego, La Jolla, CA 92093 USA; 5 Center for Virus Research, University of California, Irvine, CA 92697 USA

## Abstract

**Background:**

One potential mechanism for protection from SARS-CoV-2 in children is through passive immunity via breast milk from a mother infected with the novel coronavirus. The primary objectives of this study were to establish the presence of SARS-CoV-2-specific IgA and IgG and to characterize the antigenic regions of SARS-CoV-2 proteins that were reactive with antibodies in breast milk.

**Methods:**

Between March 2020 and September 2020, 21 women with confirmed SARS-CoV-2 infection were enrolled in Mommy’s Milk. Participants donated serial breast milk samples around their time of illness. Breast milk samples were used to probe a multi-coronavirus protein microarray containing full-length and variable-length overlapping fragments of SARS-CoV-2 proteins. Samples were also tested against S and N proteins by electrochemiluminescence assay.

**Results:**

The breast milk samples contained IgA reactive with a variety of SARS-CoV-2 antigens. The most IgA-reactive SARS-CoV-2 proteins were N (42.9% of women responded to ≥1 N fragment) and S proteins (23.9% responded to ≥1 fragment of S1 or S2). IgG responses were similar. A striking observation was the dissimilarity between mothers in antibody recognition, giving distinct antibody reactivity and kinetic profiles.

**Conclusions:**

Individual COVID-19 cases had diverse and unique milk IgA profiles following the onset of symptoms.

**Impact:**

- In this observational longitudinal case series of 21 women with confirmed SARS-CoV-2 infection, IgA binding to SARS-CoV-2 proteins detected by orthologous proteome microarray and electrochemiluminescence assays was observed in >75% of women, but there was heterogeneity in which antigens and how many were reactive between women.
- Immunological profiles of protein regions recognized by each woman were distinct.
- Diverse repertoires of mucosal breast milk antibody to SARS-CoV-2 reflect heterogeneous passive transfer of maternal antibody to exposed breastfeeding infants.

## Introduction

To date, the severe acute respiratory syndrome coronavirus 2 (SARS-CoV-2) has infected over 46 million people in the United States, resulting in over 747,000 deaths.^1^ Although COVID-19, the disease caused by SARS-CoV-2, is typically mild in children compared with adults, severe disease and death have been reported in newborns, infants, and young children.^2,3^ Multisystem inflammatory syndrome in children can occur even after the resolution of infection and has disproportionately affected ethnic minority children.^4^ Therefore, protecting the vulnerable infant and toddler population from SARS-CoV-2 is critical. One potential mechanism for protection is through passive immunity via breastfeeding from a mother previously infected with SARS-CoV-2.

Breast milk contains antibodies in response to infections that provide passive immunity, along with other bioactive factors such as lactoferrin.^5–9^ Exclusive breastfeeding substantially reduces respiratory illness in infancy and beyond.^10–13^ Continuous consumption of human milk containing pathogen-specific IgA coats the mouth, throat, and gut, preventing the establishment of infection, and is a likely component in the mechanism of protection.^14^ Approximately 90% of the antibodies found in breast milk are IgA and 8% are IgM, predominately in secretory form (sIgA/sIgM), which helps protect the antibodies from the harsh environments of the infant mouth and gut. The remaining 2% of antibodies are IgG, which are derived from serum.^15^

Early in the pandemic, the presence of both IgA and IgG antibodies to the SARS-CoV-2 spike (S) protein, the receptor-binding domain (RBD) of S and the nucleocapsid (N) protein was confirmed in breast milk from two previously infected women.^16,17^ Similarly, milk from 12 of 15 infected women contained RBD-specific IgA.^18^ The largest study to date examined 37 breast milk samples from 18 infected women and found that 76% of the milk samples contained SARS-CoV-2-specific IgA and 80% had IgG.^19^ In addition, the concentrations of SARS-CoV-2 IgA were consistently higher than IgG, which confirms the earlier report of Favara et al.^17,19^ Gao et al. were the first to report the presence of SARS-CoV-2 IgM in milk samples from 3 of 4 infected women.^20^ Antibody profiles in women with varying severity of COVID-19 symptoms have not been reported. The primary objectives of this study were to establish the presence of SARS-CoV-2-specific IgA and IgG and to characterize the antigenic regions of SARS-CoV-2 proteins that react with breast milk antibodies from women with confirmed SARS-CoV-2 infection.

## Methods

### Participants and breast milk sample collection

Breast milk samples and clinical information were obtained from women participating in the Mommy’s Milk Human Milk Biorepository at the University of California, San Diego. Women residing in the US who had a confirmed SARS-CoV-2 infection by RT-PCR were enrolled. Demographics, health history, illness and exposure dates, symptoms, and SARS-CoV-2 test results were collected by telephone interview. Participants self-collected breast milk samples using a provided collection kit including instructions for expressing and storing samples. Participants who had recovered from their illness at the time of the study interview were asked to ship any frozen samples previously collected at the peak of their symptoms in addition to a fresh milk sample. Fresh samples were shipped on ice within 24 h of collection to the Biorepository and stored at −80 °C prior to shipment on dry ice to Antigen Discovery, Inc.

### Protein microarray analysis of breast milk samples

A multi-coronavirus protein microarray, produced by Antigen Discovery, Inc. (ADI, Irvine, CA, USA), included 935 full-length proteins, overlapping protein fragments and overlapping 13–20 aa peptides from SARS-CoV-2 (WA-1), SARS-CoV, Middle East respiratory syndrome coronavirus (MERS-CoV), human coronavirus (HCoV)-NL63 and HCoV-OC43. Proteins were expressed using an *Escherichia coli* in vitro transcription and translation (IVTT) system (Rapid Translation System, Biotechrabbit, Berlin, Germany). Included on the array were four structural proteins and five accessory proteins of SARS-CoV-2: spike (S, divided into S1 and S2 regions), envelope (E), membrane (M), nucleoprotein (N), open reading frames (ORFs) 3a, 6, 7a, 8 and 10. Fragments of these nine proteins were made through IVTT in 50% overlapping segments of 30 aa, 50 aa, and 100 aa. There were also structural proteins produced by IVTT for MERS-CoV, HCoV-NL63, and HCoV-OC43. Full-length SARS-CoV-2 S protein and the RBD were included as purified proteins, plus overlapping 13–20 aa peptides of the SARS-CoV (2002 SARS epidemic) structural proteins and the S proteins of MERS-CoV, HCoV-NL63 and HCoV-OC43 (Supplementary Table S1). Purified proteins and peptides were obtained from BEI Resources. SARS-CoV-2 and SARS-CoV S proteins were made in Sf9 insect cells, and SARS-CoV-2 RBD was made in HEK-293 cells. IVTT proteins, purified proteins, and peptides were printed onto microarray slides and probed with whole breast milk samples at a 1:15 dilution for detection of IgA binding, as previously described and detailed in the Supplementary materials.^21^ The most IgA-reactive breast milk sample per patient was also probed at a 1:5 dilution for detection of IgG binding.

### ECLIA analysis of SARS-CoV-2-specific IgA and IgG in breast milk

V-PLEX COVID-19 Coronavirus Panel 2 multiplex electrochemiluminescence immune assay (ECLIA) kits were purchased from Meso Scale Discovery (Rockville, MD) to measure IgA and IgG antibodies against four SARS-CoV-2 antigens in whole breast milk samples. Antigens included were: S, RBD, N-terminal domain (NTD) of S, and N. Whole breast milk samples were diluted 1:10 and 1:100 for IgG and IgA assays, accordingly. Details of the assay are included in the Supplementary materials.

### Statistics

Maternal and infant characteristics were presented as means and standard deviations. Categorical variables were expressed as counts and percentages. Missing values were excluded. R version 4.1.0 was used for the description of maternal and child characteristics.

For protein array and ECLIA results, “reactive antigens” were defined, post hoc after observing the heterogeneity in mothers’ responses and modeling negative and positive signal distributions using mixture models (Supplementary Fig. S1).^22^ IgA reactivity for IVTT cell-free expressed proteins was defined as a normalized signal intensity greater than 1.0, equivalent to two times background, at any sample in at least one study participant. IVTT IgG reactivity was defined as normalized signal intensity greater than 2.0, or four times background. Reactivity cutoffs for array purified proteins, peptides, and ECLIA proteins for IgA and array peptides for IgG were established with the mixture models, whereas arbitrary cutoffs were set for array purified protein and ECLIA IgG signals (Supplementary Fig. S1D, H, respectively).

Clinical variables were associated with SARS-CoV-2-specific IgA antibodies using multivariable linear mixed effects regression (LMER) to model antibody responses to each SARS-CoV-2 protein or fragment with random intercepts at the subject level to adjust for repeated measures. LMER models were fit with clinical factors at the time of sample collection, including days since symptom onset, presence of COVID-19 symptoms, number of symptoms, number of days symptomatic, maternal age (years), and baby’s age (months) as fixed effects variables. All coefficients were returned from models fit using restricted maximum likelihood (ML). To generate *P* values for LMER models, the models were refit using ML and compared by ANOVA against null models with the coefficient removed using ML. For IgG responses, ordinary least squares models were fit with clinical factors, since a single sample for each study participant was assayed. *P* values were adjusted for the false discovery rate.^23^ Data visualization was performed using the circlize, ComplexHeatmap, and ggplot2 packages in R.^24–26^

## Results

### Maternal characteristics

Between March 14, 2020, and September 1, 2020, 21 women who had a confirmed SARS-CoV-2 infection by RT-PCR were enrolled. The majority of women were white (85.7%) and all non-Hispanic (Tables 1, 2). Maternal age at enrollment averaged 34.49 years (SD 3.67) and child age at enrollment averaged 10.17 months (SD 5.45). Five of the women (23.8%) had a body mass index ≥30, and 9 women (42.9%) had underlying health conditions, including asthma, hypertension, diabetes, heart conditions, kidney conditions, hypothyroidism, hyperthyroidism, or irritable bowel disease. All women were symptomatic for COVID-19, two of whom were hospitalized. An average of 9.57 (SD 4.02) symptoms were presented and lasted an average of 25.14 days (SD 15.82). Milk samples were collected at the time of onset of symptoms, and an additional one to twelve samples were taken at a range of 1–231 days post-onset of symptoms (mean number of samples collected per woman: 4.76 [SD 2.95; range 2–13]; mean number of days symptomatic: 25.14 [SD 15.82, range 10–91]). Almost one-quarter of the breastfed infants (23.8%) had respiratory symptoms, including runny nose, congestion, and fever, but were not tested for SARS-CoV-2. These infants were dyads of mothers #22, #42, #48, #60, and #63, all of whom recovered at home without medical treatment. Three (18.8%) asymptomatic infants were tested for SARS-CoV-2 and one child, the infant of mother #14, was positive.

**Table 1:** Characteristics of women who tested positive for SARS-CoV-2 and their breastfed children, *N* = 21 women and 22 children.

Select characteristics	*n* (%)
Maternal age, years (SD)	34.49 (3.67)
Race, *n* (%)	
Caucasian	18 (85.7)
Asian	2 (9.5)
Other	1 (4.8)
Ethnicity, *n* (%)	
Non-Hispanic	21 (100.0)
US region of residence, *n* (%)	
North-East	7 (33.3)
South	5 (23.8)
Mid-West	5 (23.8)
West	4 (19.0)
BMI ≥30, *n* (%)	5 (23.8)
Underlying health condition^a^, *n* (%)	9 (42.9)
Symptomatic, *n* (%)	21 (100.0)
Hospitalized, *n* (%)	2 (9.5)
Treatment with Remdesivir, *n* (%)	1 (4.8)
Treatment with ECMO^b^, *n* (%)	1 (4.8)
Number of symptoms, mean (SD), [range]	9.57 (4.02), [1,16]
Number of days symptomatic, mean (SD), [range]	25.14 (15.82), [10, 91]
Infant age (months), mean (SD)	10.17 (5.45)
Infant sex, *n* (%)	
Male	11 (50.0)
Female	11 (50.0)
Pre-term delivery, *n* (%)	5 (23.8)

^a^Underlying health conditions included: asthma, hypertension, diabetes, heart defect/ conditions, kidney defect/ condition, hypothyroidism, hyperthyroidism, IBD/ IBS.

^b^ECMO is extracorporeal membrane oxygenation.

**Table 2:** Specialized profiles for women #14, 26, 52, 56 with unique antibody response to SARS-CoV-2.

Selected characteristics	Woman #14	Woman #26	Woman #52	Woman #56
Maternal age (years)	>35	>35	30–34	30–34
Race	Asian	Caucasian	Caucasian	Asian
Ethnicity	Non-Hispanic	Non-Hispanic	Non-Hispanic	Non-Hispanic
US region of residence	East	Mid-West	East	East
BMI ≥30	Yes	No	No	No
Underlying health condition	Yes	Yes	No	No
Pregnant at the time of diagnosis	Yes	No	No	No
Symptomatic	Yes	Yes	Yes	Yes
Hospitalized	Yes	No	No	No
Treatment received: Remdesivir	Yes	No	No	No
Treatment received: ECMO	Yes	No	No	No
Number of symptoms	12	9	4	2
Number of days symptomatic	17	13	18	55
Infant age (months)	0–3	4–6	4–6	7–9
Infant sex	Male	Female	Male	Male
Pre-term delivery	Yes	No	Yes	No
SARS-CoV-2 RNA in milk	No	Yes	No	No

### Breastfeeding mothers vary in the production of milk IgA against SARS-CoV-2 proteins and recognition of specific antigenic regions

**Fig. 1:**
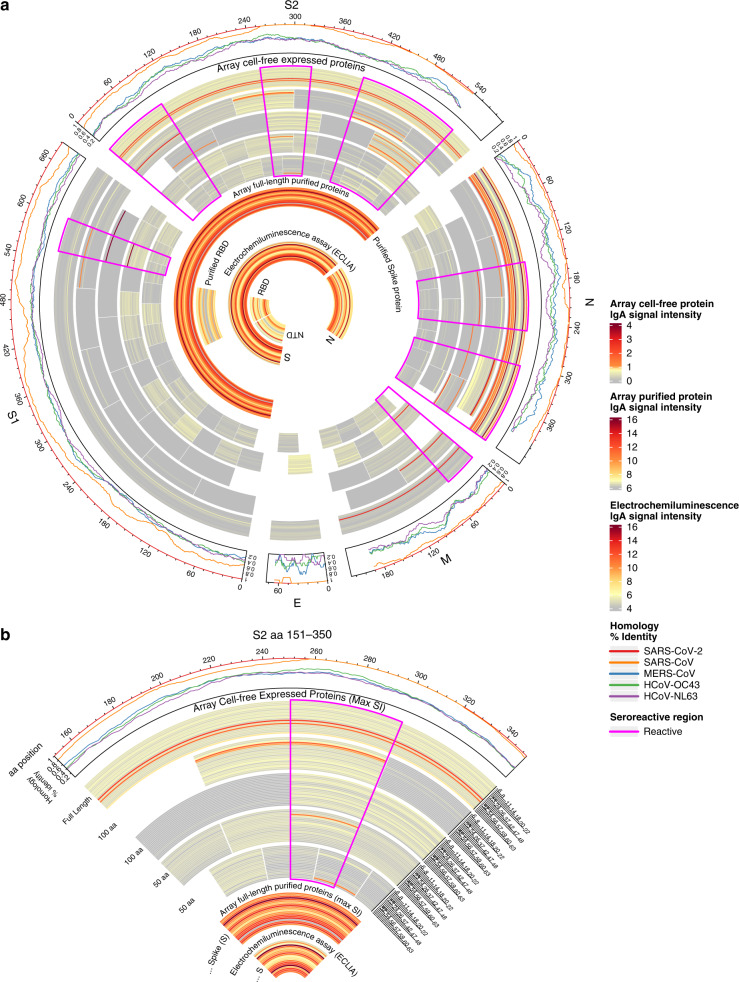
Reactivity of individual COVID-19 patient breast milk IgA to SARS-CoV-2 proteins. **a** The circular graphic maps the amino acid (aa) position of SARS-CoV-2 fragments, showing a heatmap of antibody levels for each individual mother for overlapping regions of different aa length. Proteins are indicated outside the circle plot above an axis that shows aa positions from the N-terminus to C-terminus of each protein. The following line graph shows the sequence homology of other HCoVs with SARS-CoV-2 for each gene. The inner circular heatmap shows proteins and protein fragments produced in cell-free *E. coli* in vitro transcription and translation reactions by bars that represent length and position of each fragment in each protein. Full-length, 100 aa and 50 aa fragments are shown. Fragments of 30 aa size were mostly non-reactive and are not shown but are included in the full data sets (see Supplementary Data). Each fragment is drawn 21 times, once for each COVID-19 maternal patient ordered by subject ID number, and the colored bars show the normalized signal intensity (SI) of antibody binding to each fragment. Only one data point per subject, per fragment is shown, representing the maximum SI measured among all of their respective breast milk samples taken after onset of symptoms. IgG signal intensity is shown by color gradient (gray to red). Seroreactive regions of the proteins are highlighted by magenta outlines. The inner circle bands represent the responses to full-length purified recombinant S protein (shown crossing both S1 and S2 regions) and the receptor-binding domain (RBD) of S protein from the array. This is followed by responses acquired in the electrochemiluminescence assay (ECLIA) to the full-length S and N proteins, the N-terminal domain (NTD) of S protein and the RBD of S protein. **b** The zoomed cutout of the circle graphic includes additional labeling for clarity, including fragment length labels on the left of the heatmap and subject ID labels to the right.

**Fig. 2:**
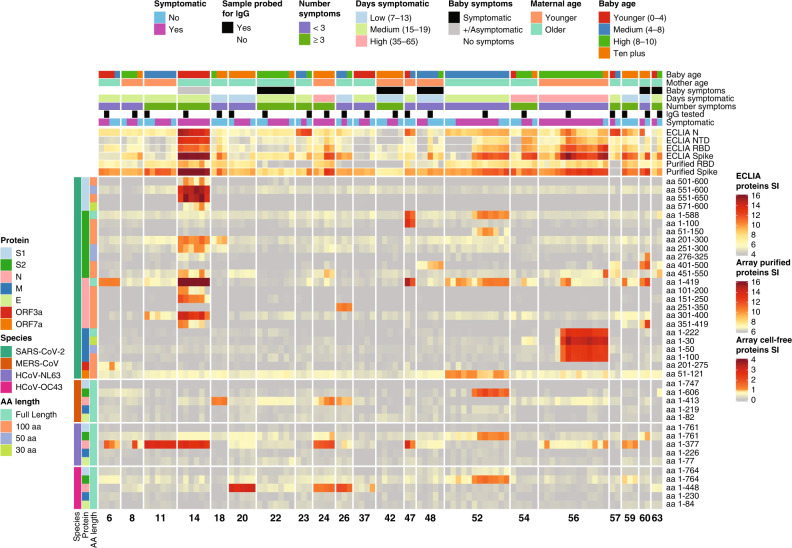
Heatmap depicting relative IgA antibody responses to SARS-CoV-2 as compared to other HCoVs and clinical data. The heatmap presents the signals of antibody binding to individual proteins and protein fragments within the antigenic regions of SARS-CoV-2, as well as the full-length structural proteins of MERS-CoV, HCoV-NL63 and HCoV-OC43, for individual samples. Columns represent breast milk samples, and rows represent proteins or protein fragments: 26 SARS-CoV-2 proteins or fragments filtered for having a maximal normalized log2 signal intensity of at least 0.5 in one or more mother’s samples, and five proteins each of MERS-CoV, HCoV-OC43 and HCoV-NL63. Antibody signal intensity is shown on a color scale from gray to red. Log2 signal intensities from recombinant purified proteins on the array and log2 signal intensity from proteins assayed on the ECLIA platform are overlaid above the array cell-free expressed proteins and shown with independent gray-to-red color scales. Sample clinical information is overlaid above the heatmaps and includes categories at time of sampling for COVID-19 symptoms, number of symptoms, number of days symptomatic, baby’s symptoms (presence or absence of respiratory symptoms, or asymptomatic if testing positive for SARS-CoV-2 “+/Asymptomatic”), maternal age and baby age. Protein/fragment information is annotated to the left of the heatmaps and includes the virus, full-length protein name and the amino acid length of the protein fragments (“AA Length”, as full length, 100, 50, or 30 aa).

Breast milk samples tested on the multi-coronavirus protein microarray (Supplementary Table S1) and by ECLIA contained IgA reactive with a variety of SARS-CoV-2 antigens, as well as antigens from other HCoVs (Figs. 1 and 2). A total of 24 IVTT-expressed SARS-CoV-2 full-length or fragmented proteins had IgA reactivity above the reactivity threshold in at least one study patient. The most IgA-reactive SARS-CoV-2 proteins were N (9/21 responded to at least one N fragment) and S proteins (5/21 for S1 or S2), and one patient exhibited strong IgA reactivity with M protein. Seropositivity rates to purified recombinant proteins on the arrays and by ECLIA were higher than for IVTT proteins, likely due to their higher concentrations: 19/21 for N by ECLIA, 18/21 for S by both protein array and ECLIA, 2/21 for the RBD by array and 16/21 by ECLIA, and 12/21 for the NTD by ECLIA. One patient responded to ORF3a, and another patient responded to ORF7a (Fig. 2). IgG seropositivity rates for IVTT proteins and array purified proteins were similar to IgA, but with more N responders (12/21), whereas N responders by ECLIA were fewer than for IgA (12/21) (Supplementary Fig. S2). These profiles are similar to our recent observations of SARS-CoV-2-specific IgG in symptomatic COVID-19 patients, where most patients responded to S and N proteins with the exception of a minor subset of “serosilent” or “serodelayed” patients.^21^^,^^27^ Moreover, the levels of reactivity seen in this study are in agreement with recent studies of maternal serum and cord blood IgG in SARS-CoV-2 infected pregnant women, as well as the reactivity of breast milk IgA from SARS-CoV-2 infected mothers with SARS-CoV-2 N, S and RBD.^19^^,^^28^

Responses to reactive regions of SARS-CoV-2 structural proteins were heterogeneous (Fig. 1). The C-terminal region of S1 spanning aa 551–650 was recognized by only mother #14 (Fig. 2). Mothers #47 and #52 had milk IgA that recognized the full-length S2 IVTT protein, but mother #47 reacted with a fragment spanning aa 1–100, whereas mother #52 reacted with a fragment spanning aa 51–150. Both mothers responded to what are likely unique epitopes within the first 150 aa of S2. Mother #14, however, responded to neither of the first 100 aa fragments of S2 nor the full-length IVTT protein, but responded specifically in the regions of aa 201–300 and aa 451–550. Mother #60 had unique IgA reactivity to the aa 401–500 fragment. For N protein, mother #14 responded to multiple fragments, whereas most N-seropositive mothers responded only to the full-length protein. Mother #26 had a seropositive IgA response only to the aa 251-350 fragment of N protein, which was non-reactive in all other mothers. The IgG levels to SARS-CoV-2 proteins were similarly heterogeneous (Supplementary Fig. S2). In another recent study, variation in the SARS-CoV-2 IgG and IgA responses in COVID-19 mRNA-vaccinated or infected pregnant and lactating women was higher in the milk than in the serum.^29^ Although antibodies were only tested against the RBD, the study by Collier et al., along with our recent profiling studies in COVID-19 patient sera, indicate that the mucosal response to SARS-CoV-2 in breast milk may be more variable than the systemic response measured in serum.^27^

### Breastfeeding mothers vary in the production of milk IgA against proteins of other human coronaviruses

All 21 of the subjects we studied had IgA antibodies that were reactive with the N and/or S proteins of one or both endemic HCoVs, HCoV-NL63, and HCoV-OC43, but the reactivity was less than two-fold over background in twelve of the mothers (Fig. 2). Ten of the patients also showed IgA reactivity with MERS-CoV N and/or S proteins. Sixteen of the patients demonstrated a likely cross-reaction of antibodies directed at SARS-CoV-2 proteins with orthologous proteins of one or more of the other three coronaviruses on the array. This is indicated by stronger reactivity with the SARS-CoV-2 protein, particularly for patients 14, 56, 59, and 6 and for patient 52 by the acquisition of strong immunity to SARS-CoV-2 midway through the time-course of sample acquisition with concomitant appearance of stronger reactivity with MERS-CoV, HCoV-NL63 and HCoV-OC43 antigens. In contrast, IgA from patients 11, 20, 24, and 26 exhibited strong reactivity with the N proteins of HCoV-NL63 and/or HCoV-OC43 without demonstrating strong reactivity with SARS-CoV-2 N. These patients likely had preexisting IgA directed to one or both HCoV and had not yet responded strongly to SARS-CoV-2.

### SARS-CoV-2 protein antibody levels were not associated with clinical factors

Our assessment of milk IgA associations with the clinical characteristics of the study patients was limited by the modest sample size of 21 women and the observed heterogeneity in IgA and IgG responses. Presence or absence of symptoms during sampling had no notable effect on IgA or IgG antibody levels (Supplementary Figs. S3 and S4). Outlier antigens had elevated IgA responses for days since onset of symptoms and days symptomatic that did not reach statistical significance after correction. These proteins included primarily fragments of S2 and N proteins, as well as the ECLIA RBD and NTD proteins (Supplementary Data). Maternal age had similar outliers, which were not significant before P-value adjustment. Other antigens, such as those associated with infant age, had low or negligible levels of IgA reactivity.

## Discussion

**Fig. 3:**
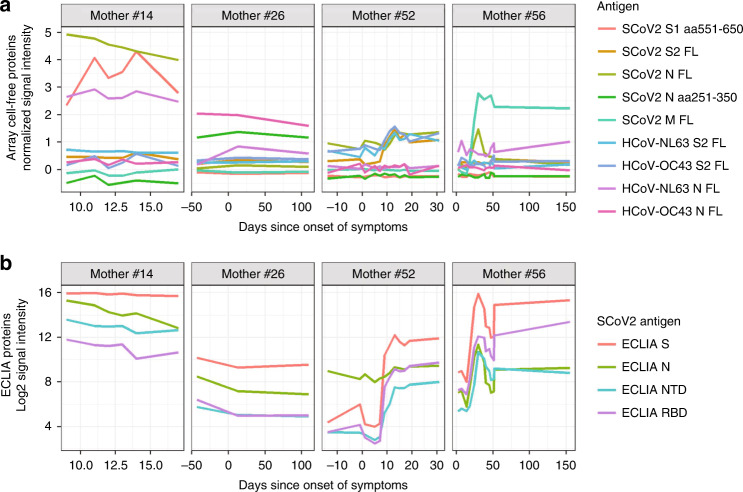
Unique longitudinal profiles of mothers’ breast milk IgA response to SARS-CoV-2 show heterogeneity in antibody recognition. **a** The line plots show breast milk IgA responses to SARS-CoV-2 and human common cold coronavirus selected full-length and protein fragments produced by the cell-free *E. coli* in vitro transcription and translation system. Antigens were selected to illustrate differences in reactivity profiles of four unique responders to SARS-CoV-2. The timing of breast milk sampling in days since onset of symptoms is shown on a free *x*-axis, and the normalized IgA signal intensity is shown in the *y*-axis. Each colored line represents an antigen’s IgA response measured for each of the longitudinal samples of one of the four mothers (each displayed in separate panels). aa amino acid, FL full-length, SCoV2 SARS-CoV-2, HCoV common cold human coronavirus. **b** The line plots show the log2 signal intensity of IgA responses to SARS-CoV-2 proteins assayed on the ECLIA platform (only SARS-CoV-2 proteins were assayed by ECLIA), where each panel is a unique mother’s responses to the four antigens. The number of days since onset of symptoms when samples were taken is shown on a free *x*-axis.

Lactating COVID-19 patients exhibited diverse and unique IgA kinetic profiles over the course of follow-up since onset of symptoms (Fig. 3). Mother #14 had the highest IgA levels among all patients, and IgA levels against both protein array antigens and ECLIA proteins were high from the initial milk sample taken. Mother #14 was the only responder to the C-terminal region of S1 spanning aa 551–650, and she was also the strongest responder to the full-length N protein. This patient tested positive for SARS-CoV-2 during the end of her pregnancy and had a pre-term delivery. The early high levels of IgA antibodies suggest an anamnestic response, perhaps due to cross-reactivity with other HCoVs. Indeed, this patient had a high response to HCoV-NL63 N protein, however, she did not respond to NL63 S2 protein. It is possible that the patient began having symptoms at a later stage of the SARS-CoV-2 infection, thereby missing longitudinal increase in antibodies.

Mother #52 exhibited a classical primary immune response with IVTT full-length S2 and N proteins, beginning low and rising at approximately 25 days post-onset of symptoms, which also tracked with NL63 and OC43 S2 proteins. For this patient, it is possible that symptoms began early in the course of SARS-CoV-2 infection. Mother #56 also had a classical primary immune response, with IgA levels peaking at around 25 days post-onset of symptoms, followed by a moderate contraction, particularly with IVTT full-length N protein, but also for the ECLIA proteins (Fig. 3) and purified S protein on the array (Fig. 2). This patient had a protracted period of symptoms, lasting 91 days. Most interestingly, mother #56 responded in the same way with very high levels of IgA to SARS-CoV-2 M protein, but not OC43 or NL63 M protein, perhaps because the immunoreactive N-terminal region of M is poorly conserved among these viruses. Another unique case was mother #26, who was not tested by PCR for SARS-CoV-2 but had a positive PCR result in a breast milk sample. She had a moderate response to SARS-CoV-2 S protein and little else, but responded specifically and solely to a fragment in SARS-CoV-2 N protein spanning aa 251-350. This patient also responded to MERS-CoV N protein and OC43 N protein (Fig. 2). Alignment of this fragment with OC43 N protein showed ~48% sequence identity between the aa 269–328 region of OC43 N protein and aa 257–319 of SARS-CoV-2 N. Alignment of the same region with NL63 N protein showed ~38% sequence identity between aa 232–307 of NL63 N protein and aa 256–331 of SARS-CoV-2 N, although there was no milk IgA reactivity against NL63 N protein. Numerous women in this study, however, responded at low levels or not at all to SARS-CoV-2 antigens with milk IgA or IgG. These patterns together with published studies on systemic immune responses to SARS-CoV-2 illustrate a complex relationship between arms of the immune system during the host response to SARS-CoV-2 infection. The causal link for why one subject responds with milk IgA to different antigenic targets than another subject remains unclear, but may be related to preferential presentation of epitopes based on MHC haplotype or other host factors.

Lack of SARS-CoV-2 testing in most of the infant dyads limited our interpretation of the effect of breast milk antibodies on infant outcomes. Notably, the infant of mother #14, who was the strongest IgA responder to spike protein, tested positive for SARS-CoV-2 and was asymptomatic. It is unknown if there were additional asymptomatic infections among the infants. However, among the five infants with symptoms, unconfirmed for SARS-CoV-2, only one was linked to a mother in the top half of responders to SARS-CoV-2 proteins on the protein array (mother #60). Further study of mothers with confirmed SARS-CoV-2 infection and close monitoring of their infants for symptomatic and asymptomatic infection is needed to examine the association of specific breast milk IgA on infant COVID-19 outcomes.

This study had several limitations. The collection of breast milk samples was not directly observed, and samples were collected with nonstandard sampling time points. Therefore, breast milk samples collected at the onset of symptoms may not reflect duration of exposure to SARS-CoV-2. We relied on maternal reports of SARS-CoV-2 test results, symptoms and treatments received, however, all participants completed a semi-structured interview guided by trained study staff who prompted for specifics with the aid of a calendar. Another limitation was lack of reactivity to the S1 protein and its fragments produced in vitro using an *E. coli*-based reaction mixture. This was likely due to the lack of eukaryotic post-translational modifications, including N-linked glycosylation which is abundant in S1. We compensated for this by including in the array purified S protein expressed in insect Sf9 cells and RBD expressed in HEK-293 cells, both systems with post-translational modifications, and by assaying purified S, RBD, and the NTD expressed in EXPi293 cells by ECLIA.

The value of this study is in the longitudinal assessment of milk antibody levels and the breadth of antigens covered by the protein microarray and ECLIA platforms, coupled with COVID-19 patient cases with unique profiles. The data show a diverse repertoire of antibody targets in SARS-CoV-2 proteins and a highly heterogeneous profile between lactating women. Thus, infants exposed to SARS-CoV-2 may benefit from breastfeeding, but they may vary in the quantity and quality of SARS-CoV-2 antibodies received.

## Supplementary information

Supplementary Material

Statistical_Analysis_PedRes_ReviewerEdits_Final

Supplementary Table S1

## Data Availability

Data access may be provided, with appropriate ethics approval, by contacting the authors.

## References

[1] Johns Hopkins University School of Medicine. COVID-19 Dashboard by the Center for System Science and Engineering (CSSE) at Johns Hopkins (accessed 4 April 2021); https://coronavirus.jhu.edu/map.html.

[2] Bhuiyan MU (2021). Epidemiology of COVID-19 infection in young children under five years: a systematic review and meta-analysis. Vaccine.

[3] Vardhelli V, Pandita A, Pillai A, Badatya SK (2021). Perinatal COVID-19: review of current evidence and practical approach towards prevention and management. Eur. J. Pediatr..

[4] Kaushik A, Gupta S, Sood M, Sharma S, Verma S (2020). A systematic review of multisystem inflammatory syndrome in children associated with SARS-CoV-2 infection. Pediatr. Infect. Dis. J..

[5] Demers-Mathieu, V. et al. Antenatal influenza A-specific IgA, IgM, and IgG antibodies in mother’s own breast milk and donor breast milk, and gastric contents and stools from preterm infants. *Nutrients*. **11**, 1567. 10.3390/nu11071567 (2019). 10.3390/nu11071567 PMC6682892 31336756

[6] Demers-Mathieu, V., Lueangsakulthai, J., Qu, Y., Scottoline, B. P. & Dallas, D. C. Binding and neutralizing capacity of respiratory syncytial virus (RSV)-specific recombinant IgG against RSV in human milk, gastric and intestinal fluids from infants. *Nutrients* **12**, 1904. 10.3390/nu12071904 (2020). 10.3390/nu12071904 PMC7400675 32605037

[7] Fouda GG (2011). HIV-specific functional antibody responses in breast milk mirror those in plasma and are primarily mediated by IgG antibodies. J. Virol..

[8] Järvinen KM, Wang J, Seppo AE, Zand M (2018). Novel multiplex assay for profiling influenza antibodies in breast milk and serum of mother-infant pairs. F1000Res..

[9] Van de Perre P (2003). Transfer of antibody via mother’s milk. Vaccine.

[10] Duijts L, Ramadhani MK, Moll HA (2009). Breastfeeding protects against infectious diseases during infancy in industrialized countries. A systematic review. Matern. Child Nutr..

[11] Henkle E (2013). The effect of exclusive breast-feeding on respiratory illness in young infants in a maternal immunization trial in Bangladesh. Pediatr. Infect. Dis. J..

[12] Schlaudecker EP (2013). IgA and neutralizing antibodies to influenza a virus in human milk: a randomized trial of antenatal influenza immunization. PLoS One.

[13] Tromp I (2017). Breastfeeding and the risk of respiratory tract infections after infancy: The Generation R Study. PLoS One.

[14] Atyeo C, Alter G (2021). The multifaceted roles of breast milk antibodies. Cell.

[15] Hurley WL, Theil PK (2011). Perspectives on immunoglobulins in colostrum and milk. Nutrients.

[16] Dong Y (2020). Antibodies in the breast milk of a maternal woman with COVID-19. Emerg. Microbes Infect..

[17] Favara DM (2020). Detection of breastmilk antibodies targeting SARS-CoV-2 nucleocapsid, spike and receptor-binding-domain antigens. Emerg. Microbes Infect..

[18] Fox A (2020). Robust and specific secretory IgA against SARS-CoV-2 detected in human milk. iScience.

[19] Pace, R. M. et al. Characterization of SARS-CoV-2 RNA, antibodies, and neutralizing capacity in milk produced by women with COVID-19. *mBio* **12**, e03192–20. 10.1128/mBio.03192-20 (2021). 10.1128/mBio.03192-20 PMC7885115 33563823

[20] Gao X (2020). Clinical and immunologic features among COVID-19-affected mother-infant pairs: antibodies to SARS-CoV-2 detected in breast milk. N. Microbes N. Infect..

[21] Camerini D (2021). Mapping SARS-CoV-2 antibody epitopes in COVID-19 patients with a multi-coronavirus protein microarray. Genomics Proteomics.

[22] Benaglia, T., Chauveau, D., Hunter, D. R. & Young, D. S. mixtools: an R package for analyzing mixture models. *J. Stat. Softw.* **32**, 1–29. 10.18637/jss.v032.i06 (2009).

[23] Benjamini Y, Hochberg Y (1995). Controlling the false discovery rate: a practical and powerful approach to multiple testing. J. R. Stat. Soc. Ser. B (Methodol.)..

[24] Gu Z, Eils R, Schlesner M (2016). Complex heatmaps reveal patterns and correlations in multidimensional genomic data. Bioinformatics.

[25] Gu Z, Gu L, Eils R, Schlesner M, Brors B (2014). Circlize implements and enhances circular visualization in R. Bioinformatics.

[26] Wickham, H. *Ggplot2: Elegant Graphics for Data Analysis* (Springer-Verlag, 2016)..

[27] Sasson JM (2021). Diverse humoral immune responses in younger and older adult COVID-19 patients. mBio.

[28] Edlow AG (2020). Assessment of maternal and neonatal SARS-CoV-2 viral load, transplacental antibody transfer, and placental pathology in pregnancies during the COVID-19 pandemic. JAMA Netw. Open..

[29] Collier, A.-R. Y. et al. Immunogenicity of COVID-19 mRNA vaccines in pregnant and lactating women. *JAMA* **325**, 2370–2380. 10.1001/jama.2021.7563 (2021). 10.1001/jama.2021.7563 PMC8120446 33983379

