# Supplementary material for: Characterization of SARS-CoV-2 antibodies in human milk from 21 women with confirmed COVID-19 infection
Source: Pediatr Res. 2022 Nov 25;93(6):1626–33. doi: 10.1038/s41390-022-02360-w (PMC9702863; doi:10.1038/s41390-022-02360-w)
Supplement: Supplementary file 1 — Supplementary Material [file 41390_2022_2360_MOESM1_ESM.pdf]

Characterization of SARS CoV-2 Antibodies in Human Milk from 21 Women with Confirmed COVID-19 Infection

Supplemental Material

Additional details for Methods

Protein microarray analysis of breast milk samples. Proteins expressed using an *E. coli* cell-free *in vitro* transcription and translation (IVTT) system, purified proteins and peptides were printed onto nitrocellulose-coated glass AVID slides (Grace Bio-Labs, Inc., Bend, OR, USA) using an Omni Grid Accent robotic microarray printer (Digilabs, Inc., Marlborough, MA, USA). Microarrays were probed with breast milk at a 1:15 dilution factor and antibody binding detected by incubation with Cy3 fluorochrome-conjugated goat anti-human IgA (Cat# 109-166-011, Jackson ImmunoResearch, West Grove, PA, USA). The rationale for selection of the 1:15 dilution factor instead of 1:5 as previously reported[21], was that higher signal-to-noise was observed for all antigens above the seropositivity threshold of 1.0 (**Supplemental Figure 5**). The most IgA-reactive breast milk sample per patient was also probed at a 1:5 dilution factor for detection of IgG binding using DyLight650 fluorochrome-conjugated goat anti-human IgG (Cat# A80-104D5, Bethyl Laboratories, Inc., Montgomery, TX, USA). Slides were scanned on a GenePix 4300A High-Resolution Microarray Scanner (Molecular Devices, Sunnyvale, CA, USA), and raw spot and local background fluorescence intensities, spot annotations and sample phenotypes were imported and merged in R (R Core Team, 2017), in which all subsequent procedures were performed. Foreground spot intensities were adjusted by subtraction of local background, and negative values were converted to one. All foreground values were transformed

using the base two logarithm. The dataset was normalized to remove systematic effects by subtracting the median signal intensity of all IVTT spots for each sample. With the normalized data, a value of 0.0 means that the intensity is no different than the background, and a value of 1.0 indicates a signal twice the background, with each subsequent unit increase representing a doubling of signals with respect to background. For full-length purified recombinant proteins and peptide libraries, the raw signal intensity data was transformed using the base two logarithm for analysis.

ECLIA analysis of SARS-CoV-2-specific IgA and IgG in breast milk. V-PLEX COVID-19 Coronavirus Panel 2 multiplex electrochemiluminescence (ECLIA) serology kits were purchased from Meso Scale Discovery (Rockville, MD) to measure Immunoglobulin A and G (IgA and IgG) antibodies against SARS-CoV-2 S, RBD, N-terminal domain (NTD) of S, and N proteins in whole breast milk samples. Pre-coated ECLIA plates were blocked for 1 h at room temperature (RT) while shaking with 150 µL Blocker A solution per well (Blocker A solution was prepared according to manufacturer instructions). Plates were washed thrice with 1x MSD Wash Buffer. A 7-point calibration curve with 4-fold serial dilutions and zero calibrator was prepared using stock calibrators using provided Diluent 100. Serum-based reference standard 1, provided with the kits, was used to establish assay calibration curves for each of the antigens. Three control samples, each containing assigned concentrations of each of the antigens were provided by the manufacturer for plate-to-plate assay validation. Whole breast milk samples were diluted 1:10 and 1:100 in 1x PBS for IgG and IgA assays, accordingly. Plates were incubated with 50 µL of prepared calibrators, serology controls, or diluted milk samples for 1 h at RT while shaking. Plates were washed thrice with 1x MSD Wash Buffer. Plates were incubated with 150 µL of 1x

detection antibody solution containing SULFO-TAG anti-human IgA or IgG prepared with Diluent 100. Plates were washed thrice with 1x MSD Wash Buffer. 150 µL of MSD GOLD Read Buffer B was added to each well, plates read on an ECLIA MESO QuickPlex SQ 120 plate reader, and the generated data analyzed with Discovery Workbench software. ECLIA signals were transformed using the base two logarithm for analysis.

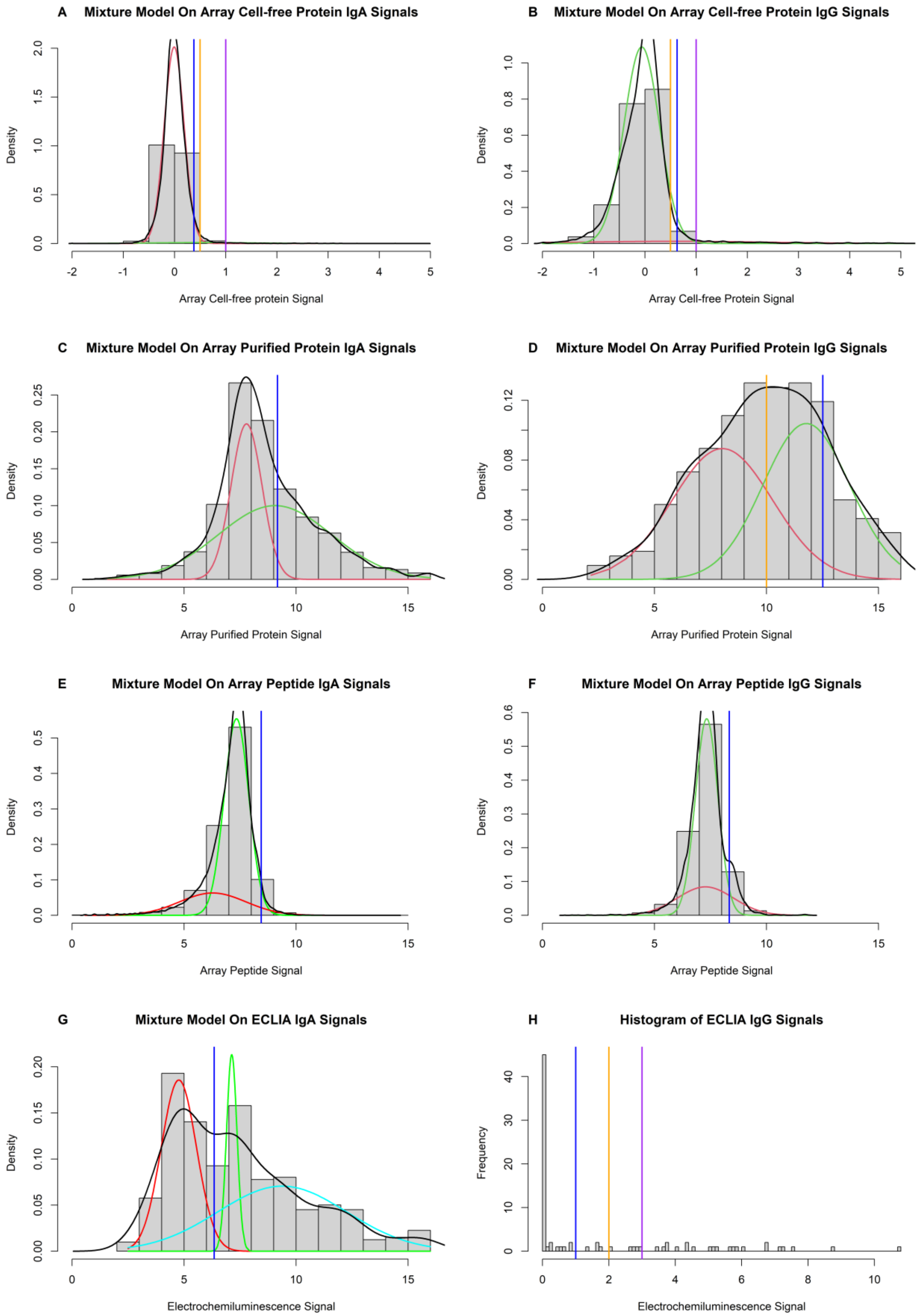
A Mixture Model On Array Cell-free Protein IgA Signals
Density
Array Cell-free protein Signal
B Mixture Model On Array Cell-free Protein IgG Signals
Density
Array Cell-free Protein Signal
C Mixture Model On Array Purified Protein IgA Signals
Density
Array Purified Protein Signal
D Mixture Model On Array Purified Protein IgG Signals
Density
Array Purified Protein Signal
E Mixture Model On Array Peptide IgA Signals
Density
Array Peptide Signal
F Mixture Model On Array Peptide IgG Signals
Density
Array Peptide Signal
G Mixture Model On ECLIA IgA Signals
Density
Electrochemiluminescence Signal
H Histogram of ECLIA IgG Signals
Frequency
Electrochemiluminescence Signal

Supplemental Figure 1. Distribution of milk IgA and IgG signals for array proteins and ECLIA proteins. (**A-G**) Mixture models were fit to antibody signals against different types of proteins on the protein microarray or ELCIA platforms to fit 2-mode Gaussian distributions, except for the ECLIA IgA signals which exhibited a trimodal distribution. Histograms and density curves (black lines) are shown in all plots. The empirically derived distribution of negative signals are shown by red lines, and the "positive" distribution by green lines, except for ECLIA IgA (**G**), which has green and cyan lines for the two positive modes. The blue vertical lines represent seropositivity cutoffs calculated from the mean and 2 standard deviations of the negative distributions. For the array cell-free expressed proteins (**A-B**), cutoffs are also shown for 0.5 (orange vertical line) and 1.0 (purple vertical line). For array purified protein IgG signals (**D**), a bimodal distribution was not observed and the mixture model did not converge, thus a line was drawn at 10.0 (orange vertical line), representing raw median fluorescence intensity (MFI) signals of approximately 1,000 and centered between the negative and positive lines. (**H**) Mixture models did not converge for ECLIA IgG data, thus a histogram is shown with cutoffs drawn at 1 (blue), 2 (orange) and 3 (purple).

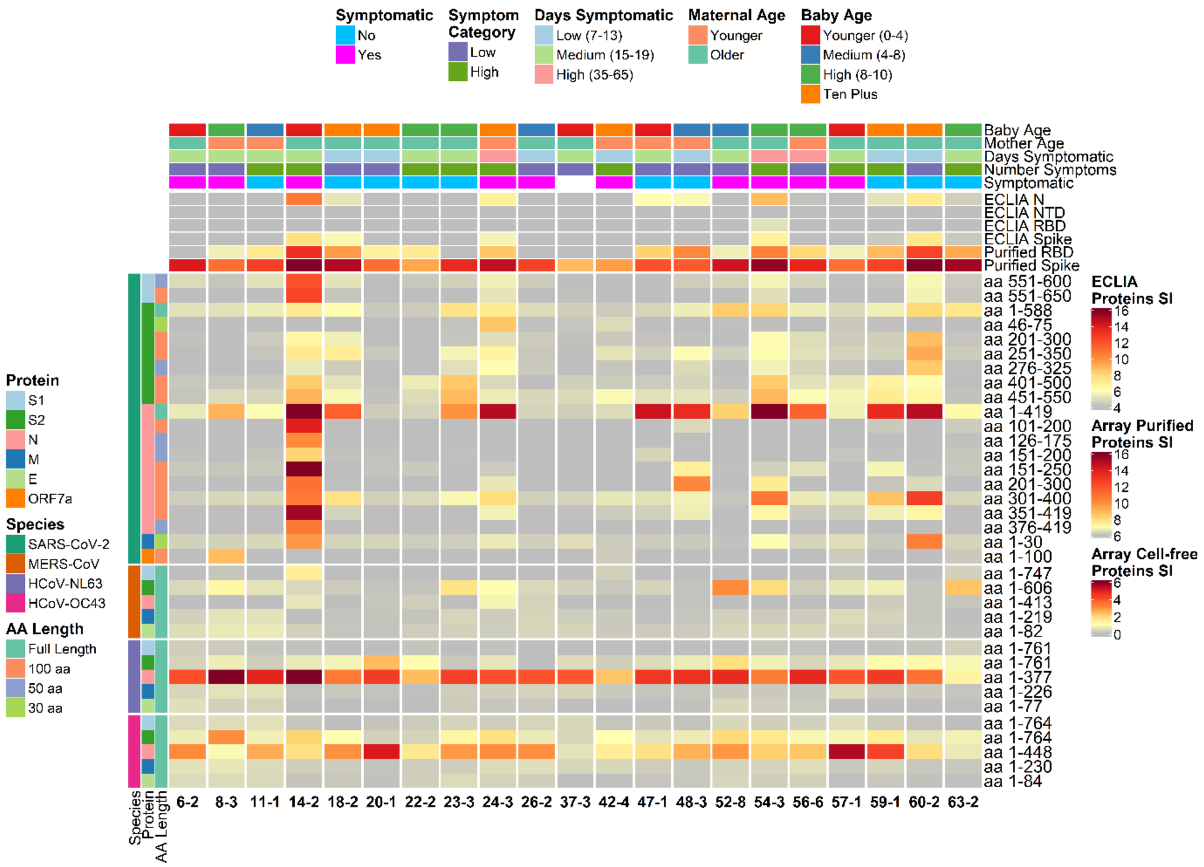

Supplemental Figure 2. Heatmap depicting relative IgG antibody responses to SARS-CoV-2 as compared to other HCoVs and clinical data. The heatmap presents the signals of antibody binding to individual proteins and protein fragments within the antigenic regions of SARS-CoV-2, as well as the full-length structural proteins of MERS-CoV, HCoV-NL63 and HCoV-OC43, for individual samples. Columns represent breast milk samples that were analyzed for IgG based on selection of one sample per mother with maximal IgA responses. Mother IDs are indicated at the bottom of each column, as well as the sample number from Supplemental Table 1 separated by "-". Rows represent proteins or protein fragments: 20 SARS-CoV-2 proteins or fragments filtered for having a maximal normalized log2 signal intensity of at least 2.0 in one or more mother's samples (noise levels for breast milk IgG were lower than IgA, and thus the normalized cutoff was set at 2.0 instead of 1.0 used for IgA), and five proteins each of MERS-CoV, HCoV-OC43 and HCoV-NL63. Antibody signal intensity is shown on a color scale from grey to red. Log2 signal intensities from recombinant purified proteins on the array and log2 signal intensity from proteins assayed on the ECLIA platform are overlaid above the array cell-free expressed proteins and shown with independent grey-to-red color scales. Sample clinical information is overlaid above the heatmaps and includes categories at time of sampling for COVID-19 symptoms, number of symptoms, number of days symptomatic, baby's symptoms (presence or absence of respiratory symptoms, or asymptomatic if testing positive for SARS-CoV-2 "+/Asymptomatic"), maternal age and baby age. Protein/fragment information is annotated to the left of the heatmaps and includes the virus, full-length protein name and the amino acid length of the protein fragments ("AA Length", as full length, 100, 50 or 30 aa).

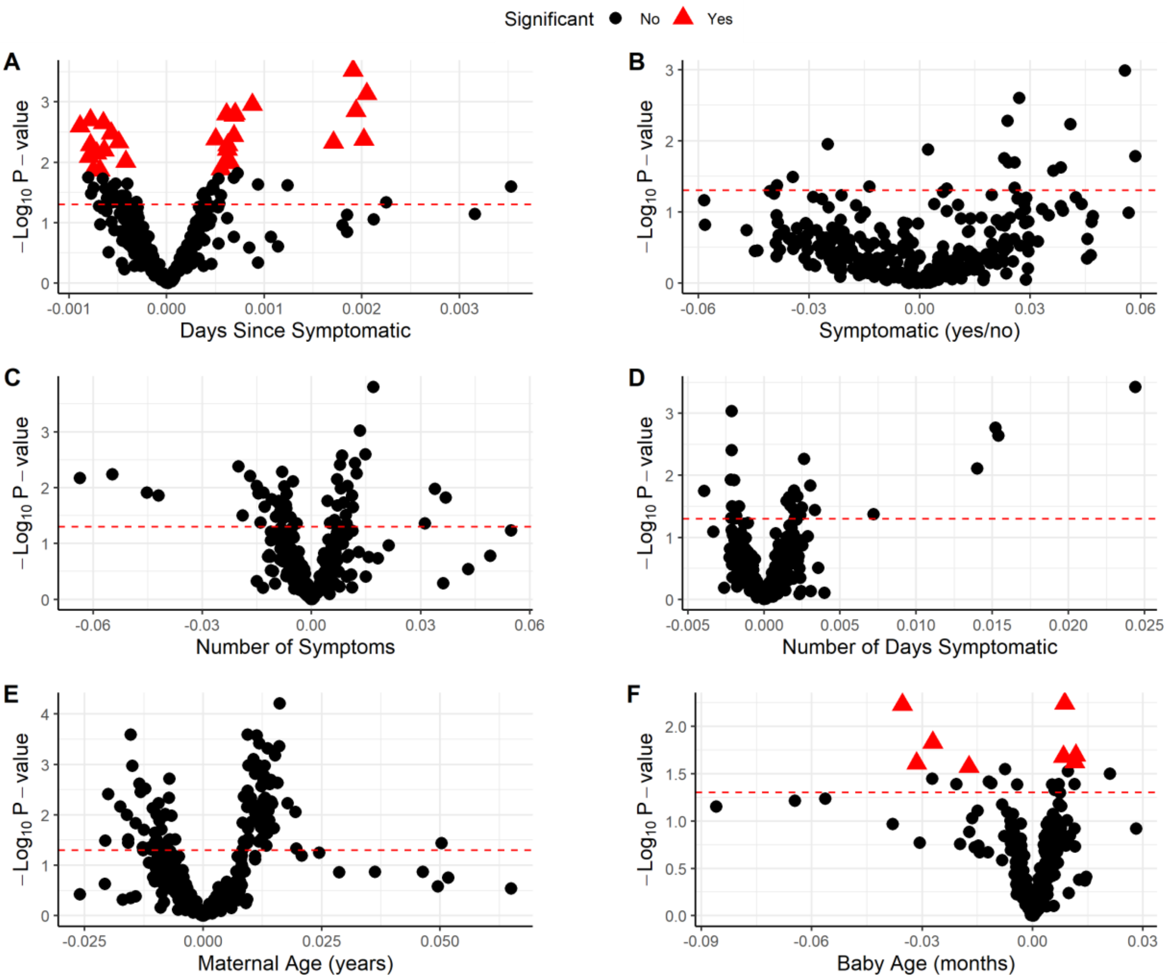

Supplemental Figure 3. Association of breast milk IgA responses to array cell-free expressed SARS-CoV-2 full length proteins and fragments with clinical factors. The volcano plots show the statistical effect estimates of clinical factors at time of sampling including (**A**) days since onset of symptoms, (**B**) presence of COVID-19 symptoms, (**C**) number of symptoms, (**D**) number of days symptomatic, (**E**) maternal age in years and (**F**) baby's age in months. The x-axis shows the linear mixed effects regression (LMER) coefficients, and the y-axis shows the inverse log10 p values for each of the SARS-CoV-2 proteins expressed using the cell-free *E. coli* expression system. The proteins/fragments with significant associations after correction for the false discovery rate are highlighted as red triangles.

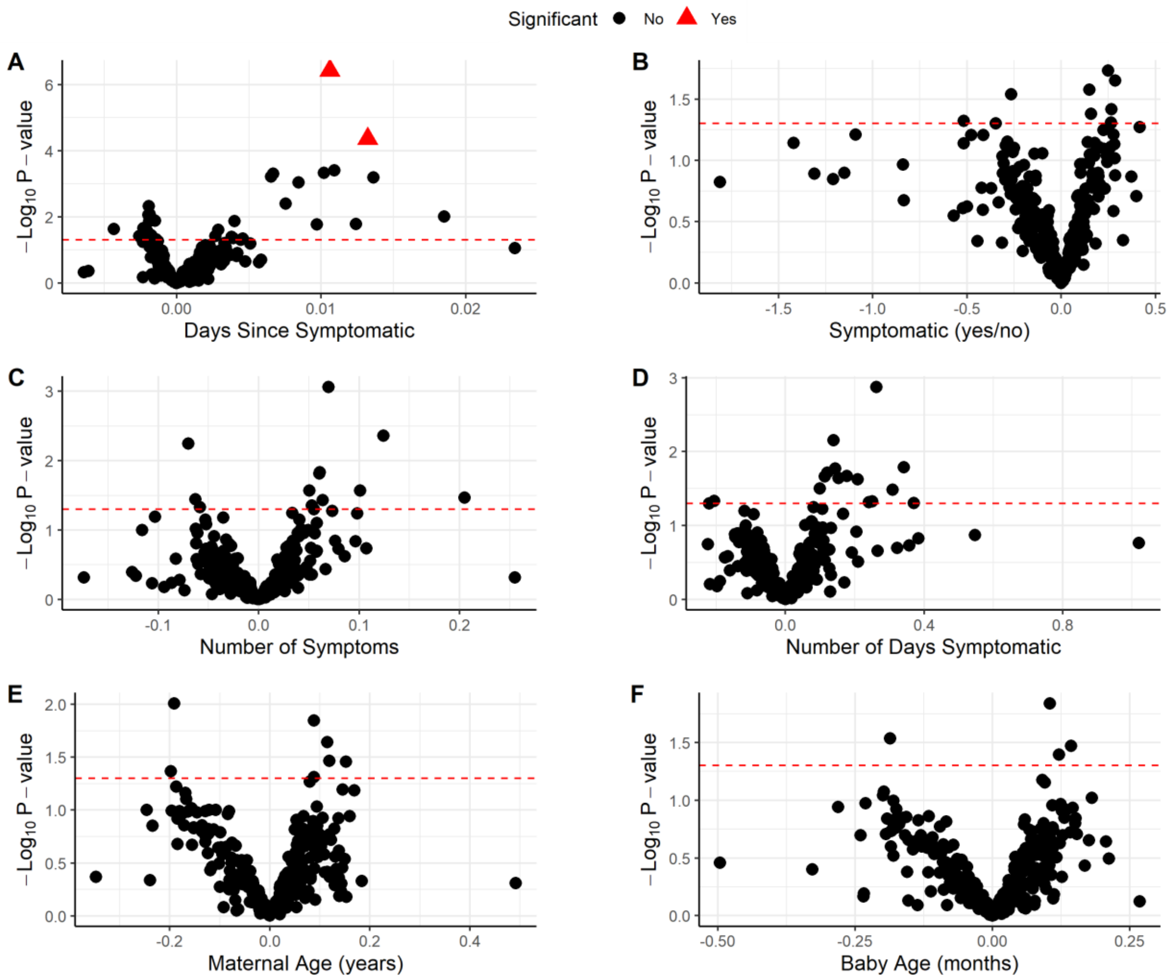

Supplemental Figure 4. Association of breast milk IgG responses to array cell-free expressed SARS-CoV-2 full length proteins and fragments with clinical factors. The volcano plots show the statistical effect estimates of clinical factors at time of sampling including (**A**) days since onset of symptoms, (**B**) presence of COVID-19 symptoms, (**C**) number of symptoms, (**D**) number of days symptomatic, (**E**) maternal age in years and (**F**) baby's age in months. The x-axis shows the ordinary least squares (OLS) regression coefficients, and the y-axis shows the inverse log10 p values for each of the SARS-CoV-2 proteins expressed using the cell-free *E. coli* expression system. The proteins/fragments with significant associations after correction for the false discovery rate are highlighted as red triangles.

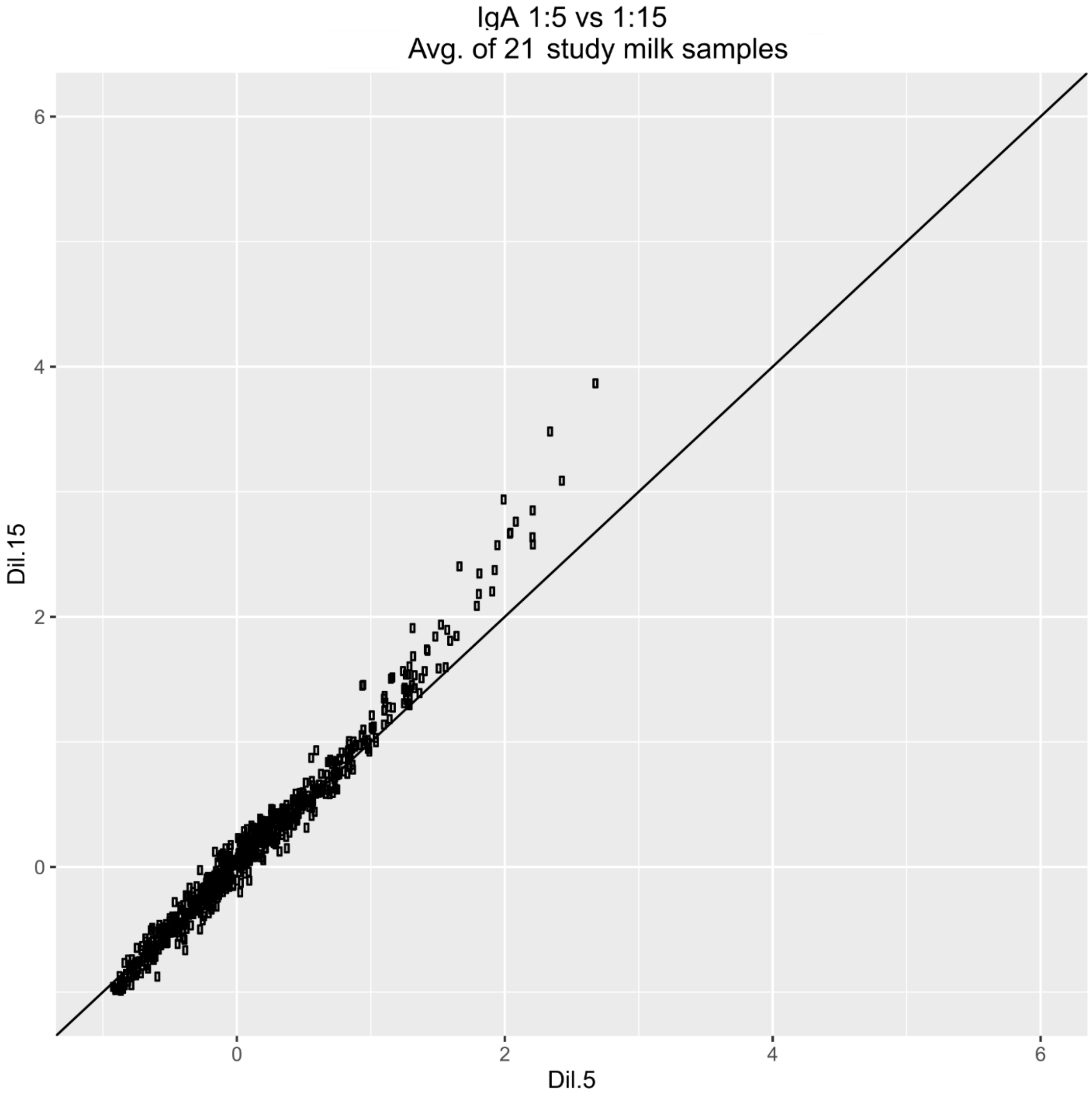

Supplemental Figure 5. Optimization of dilution factor for probing breast milk samples for IgA-only detection. The scatter plot shows the agreement of average signals from 21 breast milk samples assayed at dilution factors of 1:15 (“Dil.15”) and 1:5 (“Dil.5”). The diagonal line represents unity. Signals above the seropositivity threshold of 1.0 were higher for the 1:15 dilution factor for all antigens, likely due to better signal-to-noise ratio, and was the basis for selection of the dilution factor for the present study.
